# Biomarkers for Detecting Mitochondrial Disorders

**DOI:** 10.3390/jcm7020016

**Published:** 2018-01-30

**Authors:** Josef Finsterer, Sinda Zarrouk-Mahjoub

**Affiliations:** 1 Krankenanstalt Rudolfstiftung, Postfach 20, 1180 Vienna, Austria; 2 El Manar and Genomics Platform, Pasteur Institute of Tunis, University of Tunis, Tunis 1068, Tunisia; sinda.z.m@gmail.com

**Keywords:** biomarker, diagnosis, mitochondrial disorder, mtDNA, oxidative phosphorylation, ATP

## Abstract

(1) Objectives: Mitochondrial disorders (MIDs) are a genetically and phenotypically heterogeneous group of slowly or rapidly progressive disorders with onset from birth to senescence. Because of their variegated clinical presentation, MIDs are difficult to diagnose and are frequently missed in their early and late stages. This is why there is a need to provide biomarkers, which can be easily obtained in the case of suspecting a MID to initiate the further diagnostic work-up. (2) Methods: Literature review. (3) Results: Biomarkers for diagnostic purposes are used to confirm a suspected diagnosis and to facilitate and speed up the diagnostic work-up. For diagnosing MIDs, a number of dry and wet biomarkers have been proposed. Dry biomarkers for MIDs include the history and clinical neurological exam and structural and functional imaging studies of the brain, muscle, or myocardium by ultrasound, computed tomography (CT), magnetic resonance imaging (MRI), MR-spectroscopy (MRS), positron emission tomography (PET), or functional MRI. Wet biomarkers from blood, urine, saliva, or cerebrospinal fluid (CSF) for diagnosing MIDs include lactate, creatine-kinase, pyruvate, organic acids, amino acids, carnitines, oxidative stress markers, and circulating cytokines. The role of microRNAs, cutaneous respirometry, biopsy, exercise tests, and small molecule reporters as possible biomarkers is unsolved. (4) Conclusions: The disadvantages of most putative biomarkers for MIDs are that they hardly meet the criteria for being acceptable as a biomarker (missing longitudinal studies, not validated, not easily feasible, not cheap, not ubiquitously available) and that not all MIDs manifest in the brain, muscle, or myocardium. There is currently a lack of validated biomarkers for diagnosing MIDs.

## 1. Introduction

Mitochondrial disorders (MIDs) are metabolic disorders due to impaired metabolic pathways within mitochondria [1]. Limiting MIDs to the respiratory chain is a narrow horizon, everything that goes wrong in the mitochondrion can become a MID (e.g., a beta-oixidation defect is a MID) [1]. MIDs carry substantial morbidity and are associated with excess premature death [2]. Due to their phenotypic and genetic heterogeneity and their intra-familial and inter-familial variability, MIDs are frequently missed or wrongly diagnosed. To ascertain the suspicion of an MID, the application of simple and widely available diagnostic tests is warranted. However, there is currently a lack of validated biomarkers for diagnosing MIDs [3]. This review aims at summarising current knowledge about biomarkers in the diagnostic work-up of MIDs.

## 2. Methods

Data for this review were identified by searches of MEDLINE for references of relevant articles. Search terms used were all acronyms known for specific MIDs (*n* = 50) and the terms “mitochondrial disorder”, “mtDNA”, “encephalomyopathy”, and “mitochondrion” in individual combination with the terms “biomarker”, “diagnostic test”, “work-up”, and “diagnosis”. The results of the searches were screened for potentially relevant studies by the application of inclusion and exclusion criteria for the full texts of relevant studies. Only original articles about humans, published between 1966 and 2017, were included. Only randomised controlled trials (RCTs), observational studies with controls, case series, and case reports were included. Reviews, editorials, and letters were excluded. Additionally, reference lists of retrieved studies were checked for reports of studies not detected on the electronic search. Websites checked for additional information with regard to possible biomarkers for diagnosing MIDs were MITOMAP Neuromuscular Disease Center Database, and MitoTools.

## 3. Results

### 3.1. Biomarkers

#### 3.1.1. Definition

Biomarkers (short for biological markers) are biological measures of a biological state. By definition, a biomarker is “a characteristic that is objectively measured and evaluated as an indicator of normal biological processes, pathogenic processes or pharmacological responses to a therapeutic intervention” [4]. Thus, two main groups of biomarkers are generally differentiated: disease-related biomarkers and drug-related biomarkers [3]. Disease-related biomarkers reflect the presence or absence of disease, aid in disease stratification, guide prognosis, and can inform about disease natural history [3]. The current review focuses only on disease-related biomarkers for diagnosing or suspecting MIDs.

#### 3.1.2. Requirements

General requirements which a biomarker must meet include a continuous change of the process that is measured (changes as a function of the process being monitored), and thus a linear correlation between the measurement and the represented process, registration of quick changes (translational marker), stability without diurnal or seasonal variations, presence in detectable amounts in easily accessible biological fluids/tissues, cheap and easily feasible tests with widely available equipment, reliability when applied by different examiners and repeatedly, independence of age, sex, environmental, or climate conditions, pre-existing training condition, food, hydration, and proven usefulness, effectivity, and validation [5]. Biomarkers need to shorten the time necessary for diagnosing a condition and need to be cost-effective.

#### 3.1.3. Classification

Biomarkers can be classified according to various different criteria. In addition to separation into disease-related and drug-related biomarkers, biomarkers may be classified according to the organ or tissue they refer to or being investigated, or according to the type of tissue investigated as wet, dry, or volatile biomarkers [6]. Furthermore, biomarkers can be categorised as invasive or non-invasive or as validated or non-validated [7].

### 3.2. Dry Biomarkers

#### 3.2.1. History and Clinical Examination

Recently, an attempt has been undertaken to determine if clinical parameters from the individual or family history and findings on the clinical exam, in association with findings on easily available instrumental investigations, could raise the suspicion of an MID and could facilitate their diagnostic work-up [8]. According to this study, the so called “mitochondrial multiorgan disorder syndrome (MIMODS)” score suggests the presence of an MID if exceeding a limit of 10 points. Among 36 patients with a genetically or biochemically confirmed MID, the organs most frequently affected were the muscle (97%), the central nervous system (CNS) (72%), endocrine glands (69%), the heart (58%), intestines (55%), and the peripheral nerves (50%) [8]. MIDs manifested most frequently in the CNS as leukoencephalopathy, prolonged visually-evoked potentials or atrophy, in the endocrine organs as thyroid dysfunction, short stature, or diabetes, and in the heart as arrhythmias, heart failure, or hypertrophic cardiomyopathy [8]. Key clinical features suggesting an MID are short stature, facial dysmorphism, hypoacusis, epilepsy, migraine, cognitive impairment, diabetes, thyroid dysfunction, hypogonadism, hypertrophic cardiomyopathy, arterial hypertension, atrial fibrillation, hepatopathy, diverticulosis, nephrolithiasis, renal insufficiency, anaemia, neuropathy, and myopathy of extra-ocular, facial, bulbar, axial, respiratory, or the limb muscles. The score has not yet been validated in diseased or healthy controls. Though some MIDs may follow a pattern of organ involvement, the phenotypic heterogeneity is of such a degree that hardly a single biomarker may encompass all abnormalities developing during the course.

#### 3.2.2. Imaging

##### Structural Imaging

###### (1) Muscle

Structural alterations of the skeletal muscles in MIDs can be easily determined by ultrasound, computed tomography (CT), or magnetic resonance imaging (MRI) by measuring muscle volume, amount of connective tissues, or amount of fat. In a study of nine patients with chronic progressive external ophthalmoplegia (CPEO), the range of eye movements (ROEM) correlated with the degree of atrophy of extra-ocular eye muscles on 3 Tesla magnetic resonance imaging (3T-MRI) [9]. There was a negative correlation between ROEM and the amount of T2-hyperintensities in the extra-ocular muscles [9]. This is why the authors proposed that ROEM could serve as a marker for assessing disease severity in these patients [9]. In a study of 10 patients with CPEO due to single-scale or multiple mtDNA deletions, atrophy of extra-ocular muscles was found in all of them [10]. Though imaging with ultrasound, CT, or MRI of skeletal muscles is increasingly applied, no longitudinal studies in a large number of patients have been carried out so far.

###### (2) Brain

Structural abnormalities on imaging of the brain in patients with MIDs are manifold, and may be different between early-onset and late-onset MIDs. Structural abnormalities found in paediatric MID patients include diffuse, patchy, periventricular, subcortical, or semioval white or grey matter lesions (WMLs, GMLs), stroke-like lesions (SLLs, the morphological equivalent of stroke-like episodes, SLEs), cerebral atrophy, calcifications, or optic atrophy [11]. Some of these lesions remain stable for years, whereas others are dynamic (e.g., SLLs and GMLs) [11]. Cerebral lesions reported in adult MID patients include SLLs, laminar cortical necrosis, basal ganglia necrosis, focal or diffuse WMLs, focal or diffuse atrophy, intra-cerebral calcifications, cysts, lacunas, haemorrhages, cerebral hypo- or hyperperfusion, intra-cerebral artery stenoses, or moyamoya syndrome [12]. Since cerebral lesions in paediatric and adult MIDs may go along with or without clinical manifestations, it is important to prospectively screen patients with an MID for cerebral involvement. Most of the CNS lesions in MIDs are non-specific and are thus unsuitable for serving as biomarkers of MID with CNS involvement.

###### (3) Heart

Since the heart is frequently involved in MIDs, screening for myocardial abnormalities could be an option to prematurely detect cardiac involvement. In a study of 64 MID patients undergoing cardiac MRI, 53% had at least one cardiac abnormality [13]. Late gadolinium enhancement was found in 33%, reduced systolic function in 28%, and left ventricular hypertrophy in 22% [13]. Thickness of the left ventricular wall was generally increased in MID patients as compared to controls [13]. Among the specific MIDs, myocardial thickening was most frequent among MELAS-like patients (91% of patients), followed by patients with CPEO/ Kearns-Sayre syndrome (KSS) (late gadolinium enhancement, 80% of patients). Interestingly, more cardiac abnormalities were detected on cardiac MRI than on electrocardiogram (ECG) in this study. However, longitudinal studies to assess cardiac abnormalities over a longer period of time are warranted. 

##### Functional Imaging

###### (1) Muscle

MR-spectroscopy (MRS) of skeletal muscles in MIDs allows measurement of muscle metabolites by means of ^31^P or ^1^H spectra [3]. ^1^H-spectra reflect concentrations of lactate, choline, or N-acetyl-aspartate (NAA), whereas ^31^P spectra reflect concentrations of phosphorus metabolites and thus the oxidative capacity [3]. In the majority of cases, the phospho-creatine recovery time—which correlates with the amount of ATP production—is measured by ^31^P-MRS after phosphor-creatine depletion by exercise [3]. In a recent study on the amount of intramyocyte lipid accumulation by 7T-MRS, it turned out that the heteroplasmy rate correlated with the intramuscular lipid content in 10 patients with MELAS [14]. The authors concluded that intramyocyte lipid accumulation could serve as a novel biomarker for MELAS [14]. Another novel MRI technique allows measurement of the intramyocyte creatine content by means of creatine chemical exchange saturation transfer (CrCEST) MRI [15]. Intramyocyte creatine levels in this study correlated significantly with the capacity of oxidative phosphorylation (OXPHOS), as determined by means of ^31^P-MRS [15]. It was concluded that CrCEST allows determination of the muscle creatine content, which can be disturbed in MID patients with muscle involvement [15]. In a study of 11 patients with MELAS or CPEO, ^31^P-MRS showed an increased inorganic phosphate (iP)-to-phosphor-creatine (PCr) ratio and a decreased ATP/PCr ratio during exercise in MELAS patients [16]. Additionally, recovery to normal values of Pi/PCr and ATP/PCr was delayed in MELAS patients [16]. Energy failure as detected by ^31^P-MRS correlated with the number of COX-positive ragged-red fibres in the MELAS patients of this study [16].

###### (2) Brain

Though increased lactate production in the cerebrospinal fluid (CSF) is well appreciated among MID patients with cerebral involvement, there is little published data available investigating the capability of cerebral MRS to monitor disease progression [3]. In a study of 45 MELAS patients, the lactate peak was increased and the NAA peak was decreased compared to healthy controls [17]. Patients who developed MELAS during follow-up (converters) had elevated NAA peaks, elevated total choline peaks, elevated lactate peaks, and elevated total creatine peaks compared to healthy controls [17]. The authors concluded that CSF lactate and choline could serve as biomarkers for predicting the risk of individual mutation carriers to develop a MELAS phenotype [17]. In a study of 14 patients with nonspecific MIMODS with cerebral lesions on MRI, 86% had a lactate peak on single-voxel ^1^H-MRS of the brain [18]. Only eight patients had elevated serum lactate levels, and CSF lactate did not correlate with serum lactate [18].

Using PET studies, it has been shown that the cerebral oxygen metabolic rate is reduced, that the cerebral blood flow is increased, and that the glucose metabolic rate is increased in MELAS patients [19]. In a study of five patients with Leigh syndrome, the glucose uptake was decreased in the cerebellum and basal ganglia on 18-fluor-deoxi-glucose positron emission tomography (18FDG-PET) [20]. There are also studies which showed decreased oxygen extraction from blood during passage through the capillary bed in MID patients [21,22]. In these studies, the cerebral metabolic rate of oxygen was significantly decreased in the grey as well as the white matter in patients carrying the m.3243A>G variant, and thus it was concluded that the m.3243A>G variant results in a global decrease of oxygen consumption [22].

A promising future technique to be applied as a dry biomarker could be dynamic nuclear polarisation (DNP) MRI, which uses 13C-MRS to provide real-time functional imaging and allows determination of substrates and metabolites in low concentrations [3]. Novel PET-ligands such as 18F-BCPP-EF appear to be promising for quantification of the activity of complex-I of the respiratory chain [3].The disadvantage of all imaging biomarkers, however, is that every MID does not manifest in the brain, myocardium, or the muscle. Thus, MIDs without cerebral, myocardial, or muscle involvement may be missed by cerebral, muscle, or cardiac imaging.

#### 3.2.3. Cutaneous Respirometry 

Cutaneous respirometry is conceptualised to measure respiratory chain functions in vivo [23]. The method relies on the optical properties of proto-porphyrin-IX, a heme precursor synthesised in mitochondria, and is capable of measuring mito-pO_2_ (pO_2_: O_2_ partial pressure) and mito-VO_2_ (VO_2_: O_2_ volume) [23]. Though appealing, the method has not been applied to MIDs so far; it can be speculated that MID patients with clinical or subclinical cutaneous involvement may have increased mito-pO_2_ or increased mito-VO_2_. However, it needs to be confirmed that MID patients with involvement of the skin indeed show abnormal mito-pO_2_ or mito-VO_2_ on cutaneous respirometry. In a study of 30 healthy controls, reference limits of mito-pO_2_ are given as 44 ± 17 mm Hg, and those of mito-VO_2_ as 5.8 ± 2.3 mm Hg at 34 degrees Celsius [24]. The study showed that cutaneous respirometry allows measurement of mitochondrial oxygenation and oxygen consumption in humans [24].

### 3.3. Wet Biomarkers

#### 3.3.1. Lactate, Pyruvate, Creatine-Kinase, Amino A2cids, Organic Acids, Carnitines, Oxidative Stress Parameters

The determination of lactate, pyruvate, creatine-kinase (CK), amino acids, organic acids, carnitines, and oxidative stress parameters is frequently carried out in fluids such as blood, urine, saliva, or CSF, but diagnostic accuracy is limited. This may be due to the fact that the muscle, myocardium, and cerebrum are not affected in every MID patient, may be unaffected at the time of the investigation, and that some of these parameters depend on whether these fluids are collected at rest or during exercise. However, these parameters are attractive since the collection of appropriate fluids can be carried out non-invasively (urine, saliva) or minimally invasively (blood). Recently, it has been shown that determination of the parameters retinol-binding protein (RBP) and albumin in the urine allows the delineation of patients with specific or non-specific MIDs from healthy controls [25]. RBP (respectively, albumin) was increased in 29/75 (respectively, 23/75) patients carrying the variant m.3243A>G [25]. In a study of fibroblasts from 16 patients with Leber’s hereditary optic neuropathy (LHON) and from eight healthy volunteers, amino acids, spermidine, putrescine, isovaleryl-carnitine, propionyl-carnitine, and five sphingomyelin species were decreased, whereas ten phosphatidyl-choline species were increased [26]. Increase of sphingomyelins and decrease of phosphatidyl-choline together with decreased amino acids suggests involvement of the endoplasmic reticulum in MIDs [26].

#### 3.3.2. Circulating Cytokines (FGF21, GDF15)

The cytokines FGF-21 and GDF-15 have been recently identified as potential biomarkers of MIDs [27,28]. Since FGF-21 is mainly produced in the skeletal muscle, a main disadvantage of FGF-21 as a biomarker of MIDs is that it may not be useful in MID patients without myopathy. In MIDs which do not manifest with mitochondrial myopathy [27,28], FGF-21 may be normal. Though FGF-21 and GDF-15 have been found elevated in a significant number of MID patients, their specificity is low. This is because FGF-21 and GDF-15 have also been found elevated in other conditions, such as diabetes, hepatopathy, renal insufficiency, malignancy, or obesity [3]. Additionally, FGF-21 levels may increase with stress, steatosis hepatis, or in metabolic syndrome [29]. Whether FGF-21 levels are associated with disease severity or disease progression is under debate, since conflicting results have been reported on this issue [30,31]. Though FGF-21 concentrations correlated with disease severity in a study of 99 carriers of the m.3243A>G variant, no significant correlation was found between disease severity and the heteroplasmy rate in urinary epithelial cells or leukocytes [30]. A weak correlation was found between FGF-21 concentrations and the severity of myopathy and between FGF-21 concentrations and the severity of the encephalopathy [30]. It has recently been reported that FGF-21 and GDF-15 levels have the highest specificity in MID patients due to mtDNA translation or maintenance defects [32]. The specificity of FGF-21 (respectively, GDF-15) to detect patients with mitochondrial myopathy was 89.3% (respectively, 86.4%), and the sensitivity was 67.3% (76.0%, respectively) [32].

#### 3.3.3. microRNAs

microRNAs represent highly-conserved non-coding RNAs of 21–23 nucleotides in length (although some may reach >100 nucleotides in length), which control gene expression by silencing the transcription. Micro-RNAs regulate gene expression with high specificity on the post-transcriptional level. Micro-RNAs bind to the 3′-untranslated region (3′-UTR) of the mRNA. Due to this binding, mRNA are hindered in the translation or the mRNA is cut. Distinctive patterns of micro-RNAs are associated with various disorders and at least in cybrid cells carrying the m.3243A>G variant it has been shown that the micro-RNA 9/9* pattern is associated with the MELAS or myoclonic epilepsy with ragged-red fibers (MERRF) phenotype [33]. The micro-RNA pattern 9/9* acts as a post-transcriptional down-regulator of the mt-tRNA-modification enzymes GTPBP3, MTO1, and TRMU [33]. Down-regulation of these enzymes by microRNA-9/9* affects the U34 modification status of non-mutant tRNAs, and thus contributes to the MELAS phenotype [33].

#### 3.3.4. Biopsy of Solid Tissues

Though biopsy of affected tissues is invasive, logistically demanding, time-consuming, and cost-intensive, it is still one of the best instruments to diagnose MIDs. This is particularly the case for biopsies of the skeletal muscle, the myocardium, liver, or the skin. Biopsies can not only be analysed with regard to histological or immunohistological features, but also with regard to ultrastructural and biochemical abnormalities of mitochondria and the respiratory chain in particular. The tissue most easily accessible for biopsy is the skin. It has been shown to be of diagnostic help in Leigh syndrome with cutaneous manifestations (cutis laxa) due to a mitochondrial β-oxidation defect [34]. Skin biopsy was also of diagnostic support in a patient with MELAS and skin manifestations such as scaly, pruritic, diffuse erythema, reticular pigmentation, moderate hypertrichosis, seborrheic eczema, atopy, and vitiligo [35]. Skin biopsy may be also helpful in MIDs without skin manifestations. In MELAS patients without clinical skin manifestations, investigations of skin fibroblasts revealed decreased membrane potential of fibroblast mitochondria [36].

#### 3.3.5. Exercise Tests

Exercise tests for the diagnostic work-up of MIDs are applied to assess the aerobic capacity of mitochondria [37]. It has been shown in exercise tests of MIDs that oxygen consumption (peakVO_2_) is decreased, that peak power (Wmax) is decreased, and that also peak arterio-venous oxygen difference is decreased [37]. In a similar study, peakVO_2_ correlated with the heteroplasmy rate of the m.3243A>G variant as determined in the skeletal muscle but not in lymphocytes [38]. A second type of exercise test for diagnostic purposes is the lactate stress test [39]. It relies on the determination of serum lactate before, during, and after constant exercise below the anaerobic threshold [39]. In patients with an MID, lactate increases significantly during exercise, while in healthy subjects such an increase cannot be observed [40]. The sensitivity of the lactate stress test was calculated as 66%, and the specificity as 84% [39]. However, it is under debate as to whether the workload used should be adapted to the maximal individual workload, and if constant or incremental exercise should be carried out during the test. 

#### 3.3.6. Small Molecule Reporters 

Small molecule reporters are tailor-made probes administered intravenously to react with a substrate of interest and consecutively accumulate in mitochondria of an organ of interest [3]. After reaction with the substrate, probes are modified such that they produce an exogenous marker, which can be extracted to undergo quantitative analysis. Exogenous markers also allow inferences about the reacting substrate [3]. In a cell study, it has been shown that concentrations of reactive oxidative species (ROS) such as H_2_O_2_ can be quantified by the so-called SNAP-tag technique, which relies on the small molecule reporter SNAP-peroxy-green [41]. Though small-molecule reporters have not been applied to MID patients thus far, they may enable the measurement of mitochondrial function, mitochondrion-specific metabolites, and the generation of ROS in vivo [3].

## 4. Conclusions

This review shows that there is currently no single biomarker available with which all different subtypes of MIDs could be detected. Particularly MIDs without cerebral, cardiac, or skeletal muscle involvement may be missed by application of the biomarkers presented above. The best “biomarker” for suspecting and diagnosing MIDs is still the individual and family history and the clinical exam. Since MIDs are frequently multisystem diseases, it is essential that all organs potentially affected in an MID are prospectively investigated, irrespective of whether there are clinical manifestations. However, if patients predominantly present with cerebral, muscle, or cardiac manifestations, imaging techniques can be helpful to confirm the suspicion of an MID. Concerning the wet biomarkers from blood, urine, saliva, or CSF, lactate, pyruvate, CK, amino acids, carnitines, and organic acids are frequently elevated in various MIDs, but do not meet all criteria to serve as a biomarker. This is also the case for circulating cytokines (FGF21, GDF15) and markers of oxidative stress. Biopsies from various tissues can be extremely helpful, but are usually invasive and cost-intensive, thus not fulfilling two main criteria of a biomarker. The role of exercise tests, microRNAs, and small-molecule reporters need to be further evaluated before a decision about their role in the work-up of MIDs can be finally made. Generally, there are few biomarkers reported which have been systematically investigated for their suitability to serve as a biomarker for diagnosing an MID. Currently-available biomarkers are not appropriate to distinguish between primary and secondary MIDs. Since most of the parameters so far applied to screen for MIDs failed to meet the criteria for a biomarker, effort needs to be increased to find more global MID parameters encompassing all subtypes of a MID.
